# Association of *bla*_VIM-2_, *bla*_PDC-35_, *bla*_OXA-10,_ *bla*_OXA-488_ and *bla*_VEB-9_ β-Lactamase Genes with Resistance to Ceftazidime–Avibactam and Ceftolozane–Tazobactam in Multidrug-Resistant *Pseudomonas aeruginosa*

**DOI:** 10.3390/antibiotics11020130

**Published:** 2022-01-19

**Authors:** Mazen A. Sid Ahmed, Faisal Ahmad Khan, Hamad Abdel Hadi, Sini Skariah, Ali A. Sultan, Abdul Salam, Abdul Latif Al Khal, Bo Söderquist, Emad Bashir Ibrahim, Ali S. Omrani, Jana Jass

**Affiliations:** 1 Department of Laboratory Medicine and Pathology, Microbiology Division, Hamad Medical Corporation, Doha 3050, Qatar or mothannadona10@hotmail.com (M.A.S.A.); EElmagboul@hamad.qa (E.B.I.); 2 The Life Science Centre—Biology, School of Science and Technology, Orebro University, 701 82 Örebro, Sweden; faisal-ahmad.khan@oru.se; 3 Communicable Diseases Center, Hamad Medical Corporation, Doha 3050, Qatar; habdelhadi@hamad.qa (H.A.H.); aalkhal@hamad.qa (A.L.A.K.); aomrani@hamad.qa (A.S.O.); 4 Division of Infectious Diseases, Department of Medicine, Hamad Medical Corporation, Doha 3050, Qatar; 5 Department of Microbiology and Immunology, Weill Cornell Medicine-Qatar, Doha 2713, Qatar; sis2013@qatar-med.cornell.edu (S.S.); als2026@qatar-med.cornell.edu (A.A.S.); 6 Department of Epidemiology and Biostatistics, King Fahad Specialist Hospital, Dammam 31444, Saudi Arabia; abdul.or.salam@gmail.com; 7 School of Medical Sciences, Faculty of Medicine and Health, Orebro University, 701 82 Örebro, Sweden; bo.soderquist@oru.se

**Keywords:** *P. aeruginosa*, β-lactamases, ceftazidime–avibactam, ceftolozane–tazobactam, antimicrobial resistance, PDC-35, VEB-9

## Abstract

Ceftazidime–avibactam and ceftolozane–tazobactam are approved for the treatment of complicated Gram-negative bacterial infections including multidrug-resistant (MDR) *Pseudomonas aeruginosa*. Resistance to both agents has been reported, but the underlying mechanisms have not been fully explored. This study aimed to correlate β-lactamases with phenotypic resistance to ceftazidime–avibactam and/or ceftolozane–tazobactam in MDR-*P. aeruginosa* from Qatar. A total of 525 MDR-*P. aeruginosa* isolates were collected from clinical specimens between 2014 and 2017. Identification and antimicrobial susceptibility were performed by the BD Phoenix^TM^ system and gradient MIC test strips. Of the 75 sequenced MDR isolates, 35 (47%) were considered as having difficult-to-treat resistance, and 42 were resistant to ceftazidime–avibactam (37, 49.3%), and/or ceftolozane–tazobactam (40, 53.3%). They belonged to 12 sequence types, with ST235 being predominant (38%). Most isolates (97.6%) carried one or more β-lactamase genes, with *bla*_OXA-488_ (19%) and *bla*_VEB-9_ (45.2%) being predominant. A strong association was detected between class B β-lactamase genes and both ceftazidime–avibactam and ceftolozane–tazobactam resistance, while class A genes were associated with ceftolozane–tazobactam resistance. Co-resistance to ceftazidime–avibactam and ceftolozane–tazobactam correlated with the presence of *bla*_VEB-9_, *bla*_PDC-35_, *bla*_VIM-2_, *bla*_OXA-10_ and *bla*_OXA-488_. MDR-*P. aeruginosa* isolates resistant to both combination drugs were associated with class B β-lactamases (*bla*_VIM-2_) and class D β-lactamases (*bla*_OXA-10_), while ceftolozane–tazobactam resistance was associated with class A (*bla*_VEB-9_), class C (*bla*_VPDC-35_), and class D β-lactamases (*bla*_OXA-488_).

## 1. Introduction

Gram-negative bacteria (GNB) represent a major healthcare burden due to their association with a variety of community and healthcare-associated infections (HAIs) [1,2]. Multidrug-resistant (MDR) *Pseudomonas aeruginosa* is a challenging cause of HAIs given the limited effective treatment options, increased morbidity and mortality, as well as the cost of medical care [3,4]. Although pathogens have multiple mechanisms of resistance, it has been established that β-lactamase genes are the cornerstone of antimicrobial resistance (AMR), particularly in GNB [5]. To overcome these challenges, parallel critical measures are needed, including the prevention of AMR spread, while simultaneously developing new therapeutic modalities [3,6].

Ceftazidime–avibactam and ceftolozane–tazobactam have been approved by the United States Food and Drug Administration (US-FDA) and the European Medicines Agency (EMA) for the treatment of complicated infections caused by GNB, including ventilation-associated pneumonia, urinary tract and intra-abdominal infections [7]. The broader activity of ceftazidime–avibactam has been attributed to the addition of avibactam, a non-β-lactam β-lactamase inhibitor capable of inhibiting class A, class C, and most class D β-lactamases [8], whereas ceftolozane is a novel cephalosporin demonstrating favorable activity against *P. aeruginosa* isolates with AmpC hyper-production and overexpressed efflux mechanisms [9]. The addition of tazobactam to ceftolozane extended its activity against many, but not all, extended-spectrum β-lactamase (ESBL)-producing GNB [10].

Despite the initial remarkable success, ceftazidime–avibactam and ceftolozane–tazobactam resistance are being increasingly reported in MDR-GNB including *P. aeruginosa* [11,12]. A previous in vitro study from Qatar evaluated the efficacy of ceftazidime–avibactam and ceftolozane–tazobactam against MDR-*P. aeruginosa* isolates and reported that AMR for both agents was higher in Qatar compared to other regions worldwide [13]. The underlying mechanisms of resistance to these agents have not been fully explored. The present study aimed to characterize the β-lactamases and identify their potential correlations to phenotypic resistance of ceftazidime–avibactam and/or ceftolozane–tazobactam in MDR-*P. aeruginosa* from Qatar.

## 2. Methods

Ethical approval for the study (protocol number IRGC-01-51-033) was obtained from the Institutional Review Board at the Medical Research Council, HMC, Qatar, which complies with international ethical standards. Bacterial samples were collected prospectively between 2014 and 2017 from various clinical specimens received at the Microbiology Division of the Department of Laboratory Medicine and Pathology (DLMP), Hamad Medical Corporation (HMC), Qatar, as part of the routine care. MDR-*P. aeruginosa* was identified and subsequently stored at −80 °C for further molecular analysis. MDR-*P. aeruginosa* was defined as resistant to at least one agent from three or more different antimicrobial classes [14], whereas difficult-to-treat resistance (DTR) was defined as non-susceptibility to all first-line agents, including β-lactams, carbapenem, monobactam and fluoroquinolones [15]. A total of 525 MDR-*P. aeruginosa* were tested with ceftazidime–avibactam and ceftolozane–tazobactam, of which 75 isolates were randomly selected and subsequently processed for whole-genome sequencing (WGS), including 42 that were resistant to ceftazidime–avibactam and/or ceftolozane–tazobactam.

### 2.1. Bacterial Identification and Antimicrobial Susceptibility Testing

Bacterial identification and initial antimicrobial susceptibility tests (AST) of *P. aeruginosa* species were performed using the BD Phoenix^TM^ automated system, and identification was confirmed using matrix-assisted laser desorption ionization time of flight mass spectrometry (MALDI-TOF MS, Bruker Daltonics MALDI Biotyper, Billerica, MA, USA), according to the manufacturer’s instructions. The minimum inhibitory concentrations (MICs) were determined using Liofilchem^®^ MIC Test Strips (Liofilchem, Rosetodegli Roseto Degli Abruzzi, Italy), and the results were interpreted using the Clinical and Laboratory Standards Institute (CLSI) reference breakpoints [16]. The standard reference strains, *Escherichia coli* ATCC 25922, *E. coli* ATCC 35218, and *P. aeruginosa* ATCC 27853, were used for quality control.

### 2.2. Genomic and Phylogenetic Analyses

Seventy-five MDR-*P. aeruginosa* isolates were sent to Eurofins Genomics (GATC Biotech GmbH, Konstanz, Germany) for WGS using the Illumina HiSeq 2000 system (Illumina, San Diego, CA, USA). Following quality control assessment, trimmed reads were assembled using SPAdes, Version 3.13.0. [17]. Multilocus sequence typing (MLST) based on the seven housekeeping genes of *P. aeruginosa* isolates was performed on the MLST server 1.8 provided by The Center for Genomic Epidemiology [18]. The Comprehensive Antibiotic Resistance Database (CARD), Version 1.2.0 (McMaster University, Hamilton, Ontario) was used to annotate the antibiotic resistance genes (ARGs) in the assembled genomes [19].

### 2.3. Statistical Analysis

Descriptive and inferential statistics were used to characterize the study samples and test hypotheses. Susceptibility patterns of MDR-*P. aeruginosa* to the tested antibiotics were presented as frequency (percentages). The association between β-lactamase classes (e.g., class A, B, C, and D) and susceptibility patterns were analyzed using Pearson Chi-square and Fisher Exact test as appropriate. Cohen’s Kappa (k) was used to measure agreement between ceftazidime–avibactam and ceftolozane–tazobactam susceptibility results. MIC values of ceftazidime–avibactam against the sequence types (STs) of 75 MDR-*P. aeruginosa* were plotted using box-plot, and the median MIC values were compared by using the non-parametric Kruskal–Wallis test. The potential association between genes and antibiotic resistance to ceftazidime–avibactam and ceftolozane–tazobactam were visualized using a correlation matrix based on presence–absence data and was constructed using pairwise Spearman’s correlation between β-lactamase genes and resistance phenotype of all the *P. aeruginosa* isolates included in the study (both susceptible and non-susceptible to ceftazidime–avibactam and ceftolozane–tazobactam). Spearman’s correlation coefficient (*p*) cut-off of ≥0.4 was considered as a statistically reliable association, while a network of associations between β-lactamase genes and resistance to ceftazidime–avibactam and ceftolozane–tazobactam was constructed in Gephi using Fruchterman Reingold layout (http://gephi.org) (accessed on 15 November 2021). The co-occurrence network was further categorized into sub-networks based on the modularity index (http://gephi.org) (accessed on 15 November 2021). A *p* value < 0.05 (two-tailed) was considered statistically significant. All statistical analyses were conducted using IBM Statistical Package for Social Sciences (SPSS) for Windows, version 25.0 (IBM Corp, Armonk, NY, USA).

## 3. Results

### 3.1. Antimicrobial Susceptibility, Clinical Source, and Distribution of MDR-P. aeruginosa Isolates

Of the 525 MDR-*P. aeruginosa* tested against ceftazidime–avibactam and ceftolozane–tazobactam, 75 isolates were sent for WGS, and of these 37 (49.3%) were resistant to ceftazidime–avibactam, 40 (53.3%) were resistant to ceftolozane–tazobactam, 35 (46.7%) were resistant to both, and 33 (44%) were susceptible to both. Five isolates were resistant to ceftolozane–tazobactam but not ceftazidime–avibactam, while two isolates were resistant to ceftazidime–avibactam but not ceftolozane–tazobactam. Thus, 42 (56%) isolates were resistant to ceftazidime–avibactam and/or ceftolozane–tazobactam. Of the 75 MDR isolates, 35 (47%) were considered to be DTR, and of the 42 isolates resistant to ceftazidime–avibactam and/or ceftolozane–tazobactam, 28 (67%) were considered to be DTR (Supplementary Table S1). Thus, 80% (28/35) of the DTR isolates were also resistant to ceftazidime–avibactam and/or ceftolozane–tazobactam. The majority of MDR-*P. aeruginosa* was isolated from critical care patients at Hamad General Hospital (38, 90.5%) and most frequently from urine (14, 33.3%), followed by skin and soft tissue (10, 23.8%), while the remaining samples were collected from other sites (18, 42.9%) (Supplementary Table S2).

### 3.2. The Frequency of β-Lactamases and Sequence Types among MDR-P. aeruginosa

The 42 MDR *P. aeruginosa* isolates that were resistant to ceftazidime–avibactam and/or ceftolozane–tazobactam belonged to 12 different STs, most frequently from ST235 (16, 38.1%) and ST357 (8, 19.0%). Almost all isolates harbored class C β-lactamases (41, 97.6%) (Table 1).

**Figure 1:**
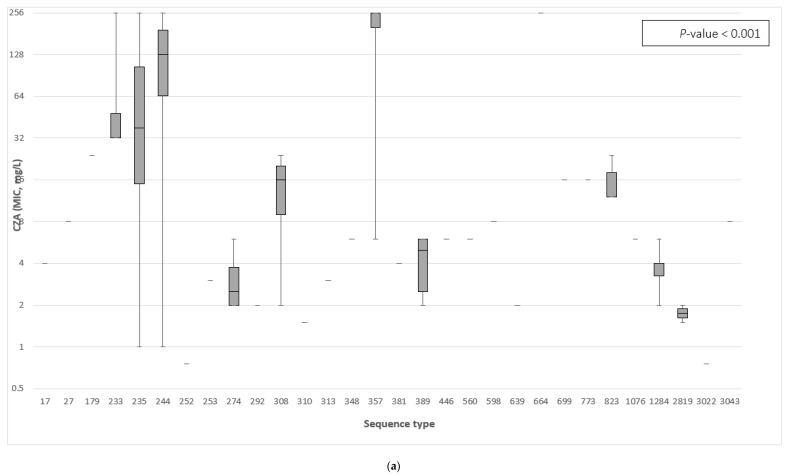

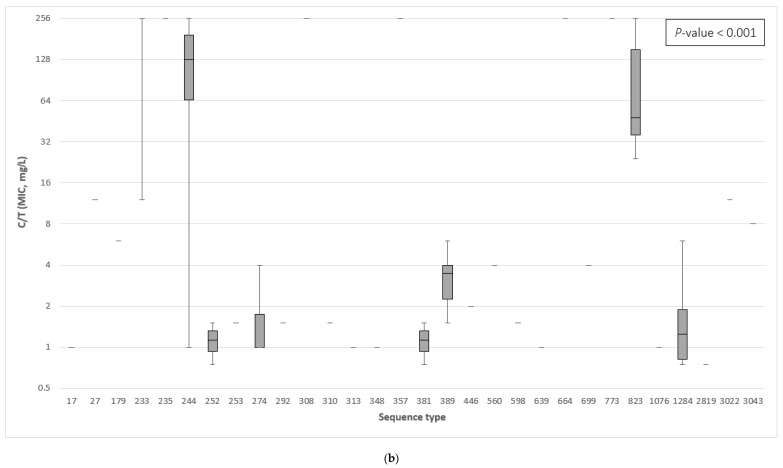
(**a**) Sequence types of MDR-*P. aeruginosa* were correlated to the MIC values of ceftazidime–avibactam (CZA). The *P*-value was measured only for those sequence types with at least two MIC values using the non-parametric Kruskal–Wallis test. All MIC values ≥256 mg/L were set to the maximum measurable MIC of 256 mg/L for this analysis. (**b**) Sequence types of MDR-*P. aeruginosa* were correlated to the MIC values of ceftolozane–tazobactam. The *p*-value was measured only for those sequence types with at least two MIC values using the non-parametric Kruskal–Wallis test. All MIC values ≥256 mg/L were set to the maximum measurable MIC of 256 mg/L for this analysis.

The distribution of the MIC values of the 75 MDR-*P. aeruginosa* tested against ceftazidime–avibactam and ceftolozane–tazobactam in relation to different ST was assessed (Figure 1a,b); the median MIC values (range) of ceftazidime–avibactam for ST235, ST244, and ST357 were 38 mg/L (1, 256), 128.5 mg/L (1, 256), and 256 mg/L (6, 256), respectively. Furthermore, the median MIC values (range) of ceftolozane–tazobactam for ST244 were 128.5 mg/L (1, 256), and for ST823 were 48 mg/L (24, 256).

**Figure 2:**
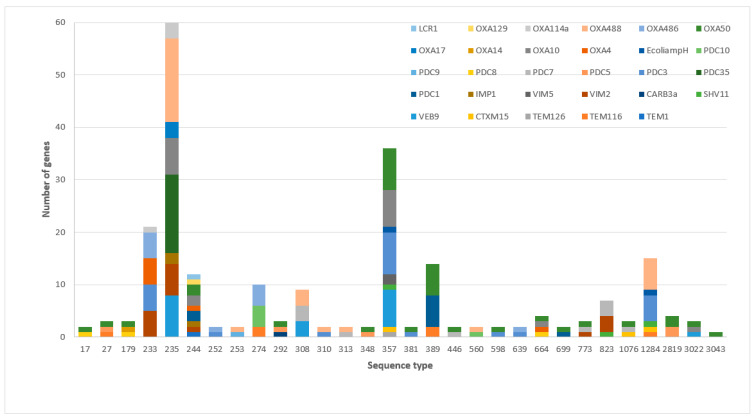
The distribution of β-lactamase genes was associated with different MDR-*P. aeruginosa* sequence types.3.3. The association of β-lactamase classes and resistance to ceftazidime–avibactam and ceftolozane–tazobactam.

Β-Lactamase genes demonstrated associations with different sequence types; class C *bla*_PDC-35_ 100% (15/15) was associated with ST235, while class A *bla*_VEB-9_, with 42.1% (8/19), 36.8% (7/19), 15.8% (3/19), and 5.3% (1/19), was associated with ST235, ST357, ST308, and ST3022, respectively. Class B *bla*_VIM-2_ was associated with ST235 at 37.5% (6/16), ST233 at 31.3% (5/16), ST823 at 18.8% (3/16), ST244, and ST773 at 6.3% (1/16), while class D *bla*_OXA-488_ was associated with ST235 at 55.2% (16/29), ST1284 at 20.7% (6/29), ST308 at 10.3% (3/29), and ST253, ST310, ST313, and ST560 at 3.4% (1/29) (Figure 2).

The presence of class B β-lactamase genes was significantly associated with resistance to ceftazidime–avibactam and ceftolozane–tazobactam (*p*-value < 0.001) in the 75 MDR-*P. aeruginosa* isolates tested. A strong association between the presence of class A and resistance to ceftolozane–tazobactam (*p*-value = 0.005) was also evident (Table 2).

### 3.3. The Association between the β-Lactamase Genes and the Minimum Inhibitory Concentration of Ceftazidime–Avibactam and Ceftolozane–Tazobactam

A total of 29 different β-lactamase genes were detected in the 75 MDR-*P. aeruginosa*. The predominant β-lactamases were *bla*_OXA-50_ 41.3% (32/75), *bla*_OXA-488_ 37.3% (29/75), *bla*_PDC-3_ 30.7% (23/75) *bla*_VEB-9_ 25.3% (19/75), and others (Table 3). A strong association was found between the completely resistant isolates displaying MIC_256_ to ceftolozane–tazobactam with the presence of *bla*_PDC-35_ at 100% (15/15), *bla*_VEB-9_ at 94.7% (18/19), *bla*_OXA-10_ at 88.9% (16/18), *bla*_VIM-2_ at 81.3% (13/16), and *bla*_OXA-488_ at 67.9% (19/28), while the resistance range MIC_12-192_ to ceftazidime–avibactam was associated with the presence of *bla*_VIM-2_ at 87.5% (14/16) and *bla*_PDC-35_ at 86.7% (13/15) (Table 3).

### 3.4. Correlation of Specific β-Lactamase Genes to Ceftazidime–Avibactam and Ceftolozane–Tazobactam Resistance

**Figure 3:**
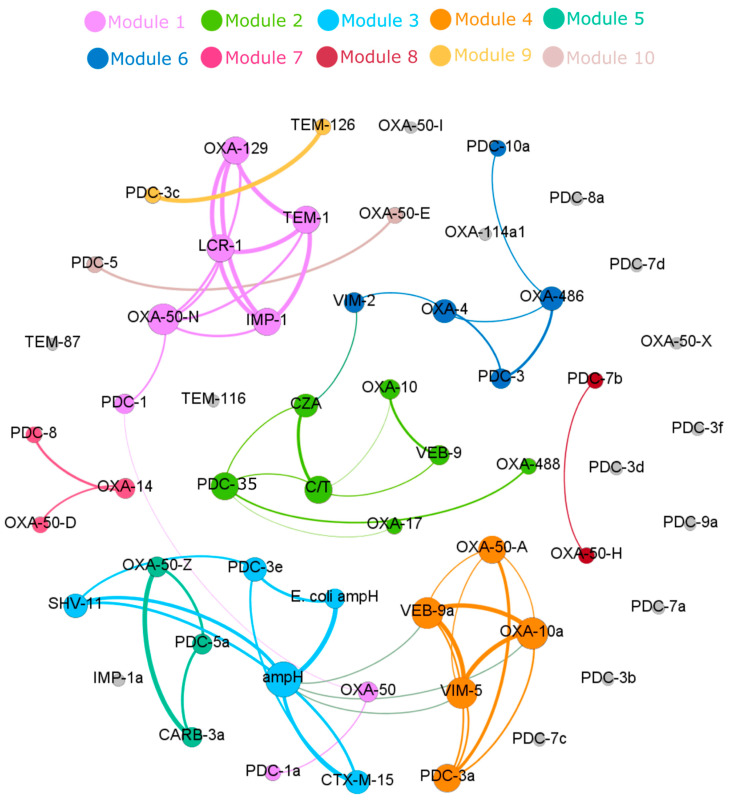
The co-occurrence network based on the Spearman’s correlation of all β-lactamase genes and resistance phenotypes in 75 MDR-*P. aeruginosa* strains, performed and visualized by Gephi network analysis (*p* ≥ 0.4). The node size represents the number of connections with other antibiotic resistance genes. The thickness of the edge is proportional to Spearman’s correlation coefficient (*p*) of the connection. The co-occurrence network is further divided into 10 subnetworks (modules) based on modularity class (modularity index = 0.477), and modules are highlighted in different colors. Genes in gray color do not show a significant correlation. β-lactamase (amino acid mutation); class CPDC-3a (P7S, G391A), PDC-3b (V205L, G391A), PDC-3c (P7S, V205L), PDC-3d (P7S, V205L, G391A), PDC-3e (G391A), PDC-3f (G229S), PDC-5 (V205L, P274L), PDC-7a (G391A), PDC-7b (I356V, G391A), PDC-7c (P7S, Q115R, G391A), PDC-7d (P7S, Q115R, I356V, G391A), PDC-9 (V27D, G391A), PDC-10a (G391A). Class D; OXA-50-M (T16A, Q25R), OXA-50-Z (R49C, D109E, A134G, R167H, A181T), OXA-50-E (D109E, R167H), OXA-50-N (R49C, D109E, R167H), OXA-50-I (T16A, R83K), OXA-50-D (R6F, D109E, R167H), OXA-50-H (T16A, Q25R, R83K). Amino acids: A: alanine; C: cysteine; D: aspartic acid; E: glutamic acid; F: phenylalanine; G: glycine; H: histidine; I: isoleucine; K: lysine; L: leucine; M: methionine; P: proline; Q: glutamine; R: arginine; S: serine; T: threonine; V: valine.

The co-occurrence network of all the β-lactamase genes and the phenotypic resistance to ceftazidime–avibactam and ceftolozane–tazobactam in 75 MDR *P. aeruginosa* is shown in Figure 3. Spearman’s correlation showed that resistance to ceftazidime–avibactam was associated with the presence of *bla*_VIM-2_ (0.53) or *bla*_PDC-35_ (0.51) in the isolates, while resistance to ceftolozane–tazobactam correlated with the presence of *bla*_VEB-9_ (0.53), *bla*_PDC-35_ (0.49), and *bla*_OXA-10_ (0.45). Co-occurrence of other β-lactamase genes did not correlate to either ceftazidime–avibactam or ceftolozane–tazobactam resistance.

## 4. Discussion

The present study reported 42 MDR-*P. aeruginosa* isolates that were phenotypically resistant to ceftazidime–avibactam and/or ceftolozane–tazobactam, and many were also considered as DTR. They belonged to 12 different STs, with a high frequency of ST235, ST357, ST233, and ST308. These notorious epidemic clones exhibiting high MIC values for ceftazidime–avibactam and ceftolozane–tazobactam are responsible for spreading resistance globally, including in the Middle East region [20,21,22]. Nearly all resistant isolates harbored at least one gene of class C and class D β-lactamases, while nearly half of the resistant isolates had class A and class B β-lactamases (Table 1). The results are comparable to other studies reported in the region [23,24]. Furthermore, our results demonstrate a significant association between the presence of a class B β-lactamase and resistance to both ceftazidime–avibactam and ceftolozane–tazobactam, while class A β-lactamase was significantly associated with resistance only to ceftolozane–tazobactam (Table 2). Similarly, it has been established that the production of class B enzymes is linked with resistance to both combinations, [25] while different class A ESBLs in *P. aeruginosa* are linked to the observed resistance to ceftolozane–tazobactam [26]. In addition, *bla*_VIM-2_ is a metallo-β-lactamase (MBL) that utilizes Zn^2+^ as a nucleophile in the active site for the hydrolysis of β-lactams [25]. β-lactamase inhibitors such as tazobactam and avibactam can inhibit class A, C, and some of class D (serine β-lactamases), but not MBL, such as *bla*_VIM-2_ [25]. This corresponds well with our results, where *bla*_VIM-2_ was detected in half of the isolates that were found to be resistant to ceftazidime–avibactam and ceftolozane–tazobactam, and *bla*_VIM-2_ and were mainly associated with ST235 and ST233.

Class A β-lactamase *bla*_VEB-9_ which was mainly associated with ST235 and ST357, was predominant in 45.2% of the resistant isolates and significantly associated with high resistance (MIC_256_) to ceftolozane–tazobactam and, to a lesser degree, ceftazidime–avibactam (Table 3). Although *bla*_VEB-9_ has been described by different regions worldwide, [27,28,29] its role in ceftolozane–tazobactam resistance warrants further exploration. It is worth highlighting that class C β-lactamase genes were detected in nearly all the resistant isolates with a predominance of ESBL, *bla*_PDC-3_, and *bla*_PDC-35_. These extended-spectrum cephalosporinases have been previously shown to be associated with ceftolozane–tazobactam resistance [30,31].

In our collection, *bla*_PDC-35_, with 99.75% resemblance to *bla*_PDC-2_, has only one amino acid substitution, glycine to alanine at position 391 (G391A) (NCBI Reference Sequence: NG_049907.1). Intriguingly, *bla*_PDC-35_ was exclusively detected in ST235 and was significantly associated with high-level resistance to ceftolozane–tazobactam (MIC_256_) and, to a lesser degree, ceftazidime–avibactam (Table 3). A similar association between *bla*_PDC-35_ and resistance to ceftolozane–tazobactam and ceftazidime–avibactam was recently reported [32]. Moreover, a previous study identified different mutations in class C AmpC, glycine to aspartate substitution in position 183 (G183D) in class C AmpC associated with a low-level of resistance to ceftolozane–tazobactam [33].

The highly prevalent Class D β-lactamase genes included *bla*_OXA-488_ (GenBank: TKV86805.1) and *bla*_OXA-486_ (GenBank: QBY97442.1), which are variants of the intrinsic oxacillinase *bla*_OXA-50_, had only two-point amino acid substitutions at position T16A and Q25R and R49C and D109E, respectively, and this necessitated their reclassification. Furthermore, *bla*_OXA-10_ and *bla*_OXA-17_, which is a variant of *bla*_OXA-10_ with substitutions of asparagine by serine at position 77, [34] were found to be associated with extreme resistance to ceftolozane–tazobactam in the present study. Conversely, *bla*_OXA-4_ was closely linked to ST233 and has only been detected in isolates resistant to both combinations, while *bla*_OXA-10_, which is mainly linked to ST235 and ST357, was almost universally resistant to ceftolozane–tazobactam and highly resistant to ceftazidime–avibactam. A recent study reported the association between *bla*_OXA-10_ derivatives and resistance to both combination drugs [35]. Furthermore, *bla*_OXA-488_ was linked to ST235 and ST1284 and has a noticeable resistance to both combinations. Despite the emergence of many new variants of the OXA-type β-lactamases, few studies have been conducted to evaluate their possible roles such as the *bla*_OXA-10_, [12], and *bla*_OXA-50_ family (i.e., *bla*_OXA-486,_ *bla*_OXA-488_) [31,36] detected in the present study. On the other hand, the mutant *bla*_OXA-4_ and the selection for an extended-spectrum *bla*_OXA-2_ derivative (*bla*_OXA-539_) results in ceftazidime–avibactam and ceftolozane–tazobactam resistance [12,37].

To the best of our knowledge, this is one of the largest collections from the Middle East, examining 75 MDR-*P. aeruginosa* by genomic characterization of resistance to ceftazidime–avibactam and ceftolozane–tazobactam. We acknowledge that a combination of multiple resistance mechanisms encompassing β-lactamases, modification of outer membrane proteins, and upregulation of efflux pumps, which was not examined here, may also contribute to the high resistance to ceftazidime–avibactam and ceftolozane–tazobactam in some of the *P. aeruginosa* isolates [21,38]. A possible study limitation may be the use of a gradient test Liofilchem MIC test strips as a standard susceptibility testing method for ceftazidime–avibactam and ceftolozane–tazobactam, since subsequent studies suggested that broth microdilution methods might be more accurate in defining resistance [36,39].

## 5. Conclusions

In conclusion, the characterization and prevalence of various β-lactamase genes in clinical MDR-*P. aeruginosa* isolates from Qatar that were phenotypically resistant to ceftazidime–avibactam and/or ceftolozane–tazobactam revealed the presence of a diverse group of β-lactamase genes. Resistance to both ceftazidime–avibactam and ceftolozane–tazobactam was associated with the presence of class B β-lactamases (e.g., *bla*_VIM-2_) and class D β-lactamases (e.g., *bla*_OXA-10_), whilst ceftolozane–tazobactam resistance was associated with class A β-lactamases (e.g., *bla*_VEB-9_), class C β-lactamases (e.g., *bla*_PDC-35_), and class D β-lactamases (e.g., *bla*_OXA-488_). The presence of other β-lactamase genes such as *bla*_PDC-35_ and *bla*_OXA-10_ also correlated with resistance to these novel therapeutic agents.

## Figures and Tables

**Table 1:** The frequency of MDR-*P. aeruginosa* sequence types associated with β-lactamase classes are categorized according to the Ambler classification and their susceptibility pattern to ceftazidime–avibactam and ceftolozane–tazobactam.

Sequence Type	No. Strains (Frequency)	β-Lactamase Class								Antimicrobial Susceptibility			
		Class A		Class B		Class C		Class D		CZA		C/T	
		No	Yes	No	Yes	No	Yes	No	Yes	R	S	R	S
235	16 (21.3)	8 (17.8)	8 (26.7)	8 (14.5)	8 (40)	1 (33.3)	15 (20.8)	0	16 (22.2)	15 (40.5)	1 (2.6)	16 (40)	0
357	8 (10.7)	1 (2.2)	7 (23.3)	6 (10.9)	2 (10)	0	8 (11.1)	0	8 (11.1)	7 (18.9)	1 (2.6)	8 (20)	0
389	6 (8)	4 (8.9)	2 (6.7)	6 (10,9)	0	0	6 (8.3)	0	6 (8.3)	0	6 (15.8)	0	6 (17.1)
1284	6 (8)	4 (8.9)	2 (6.7)	6 (10,9)	0	0	6 (8.3)	0	6 (8.3)	0	6 (15.8)	0	6 (17.1)
233	5 (6.7)	5 (11.1)	0	0	5 (25)	0	5 (6.9)	0	5 (6.9)	5 (13.5)	0	5 (12.5)	0
274	4 (5.3)	2 (4.4)	2 (6.7)	4 (7.3)	0	0	4 (5.6)	0	4 (5.6)	0	4 (10.5)	0	4 (11.4)
308	3 (3)	0	3 (10)	3 (5.5)	0	0	3 (4.2)	0	3 (4.2)	2 (5.4)	1 (2.6)	3 (7.5)	0
823	3 (4)	2 (4.4)	1 (3.3)	0	3 (15)	0	3 (4.2)	3 (100)	0	3 (8.1)	0	3 (7.5)	0
244	2 (2.7)	1 (2.2)	1 (3.3)	1 (1.8)	1 (5)	0	2 (2.8)	0	2 (2.8)	1 (2.7)	1 (2.6)	1 (2.5)	1 (2.9)
2819	2 (2.7)	2 (4.4)	0	2 (3.6)	0	0	2 (2.8)	0	2 (2.8)	0	2 (5.3)	0	2 (5.7)
17	1 (1.3)	1 (2.2)	0	1 (1.8)	0	0	1 (1.4)	0	1 (1.4)	0	1 (2.6)	0	1 (2.9)
27	1 (1.3)	0	1 (3.3)	1 (1.8)	0	0	1 (1.4)	0	1 (1.4)	0	1 (2.6)	1 (2.5)	0
179	1 (1.3)	1 (2.2)	0	1 (1.8)	0	0	1 (1.4)	0	2 (1.4)	1 (2.7)	0	0	1 (2.9)
252	1 (1.3)	1 (2.2)	0	1 (1.8)	0	0	1 (1.4)	0	1 (1.4)	0	1 (2.6)	0	1 (2.9)
253	1 (1.3)	1 (2.2)	0	1 (1.8)	0	0	1 (1.4)	0	1 (1.4)	0	1 (2.6)	0	1 (2.9)
292	1 (1.3)	0	1 (3.3)	1 (1.8)	0	0	1 (1.4)	0	1 (1.4)	0	1 (2.6)	0	1 (2.9)
310	1 (1.3)	1 (2.2)	0	1 (1.8)	0	0	1 (1.4)	0	1 (1.4)	0	1 (2.6)	0	1 (2.9)
313	1 (1.3)	1 (2.2)	0	1 (1.8)	0	0	1 (1.4)	0	1 (1.4)	0	1 (2.6)	0	1 (2.9)
348	1 (1.3)	1 (2.2)	0	1 (1.8)	0	0	1 (1.4)	0	1 (1.4)	0	1 (2.6)	0	1 (2.9)
381	1 (1.3)	1 (2.2)	0	1 (1.8)	0	0	1 (1.4)	0	1 (1.4)	0	1 (2.6)	0	1 (2.9)
446	1 (1.3)	1 (2.2)	0	1 (1.8)	0	0	1 (1.4)	0	1 (1.4)	0	1 (2.6)	0	1 (2.9)
560	1 (1.3)	1 (2.2)	0	1 (1.8)	0	0	1 (1.4)	0	1 (1.4)	0	1 (2.6)	0	1 (2.9)
598	1 (1.3)	1 (2.2)	0	1 (1.8)	0	0	1 (1.4)	0	1 (1.4)	0	1 (2.6)	0	1 (2.9)
639	1 (1.3)	1 (2.2)	0	1 (1.8)	0	0	1 (1.4)	0	1 (1.4)	0	1 (2.6)	0	1 (2.9)
664	1 (1.3)	1 (2.2)	0	1 (1.8)	0	0	1 (1.4)	0	1 (1.4)	1 (2.7)	0	1 (2.5)	0
699	1 (1.3)	1 (2.2)	0	1 (1.8)	0	0	1 (1.4)	0	1 (1.4)	1 (2.7)	0	0	1 (2.9)
773	1 (1.3)	1 (2.2)	0	0	1 (5)	0	1 (1.4)	0	1 (1.4)	1 (2.7)	0	1 (2.5)	0
1076	1 (1.3)	0	1 (3.3)	1 (1.8)	0	0	1 (1.4)	0	1 (1.4)	0	1 (2.6)	0	1 (2.9)
3022	1 (1.3)	0	1 (3.3)	1 (1.8)	0	1 (33.3)	0	0	1 (1.4)	0	1 (2.6)	1 (2.5)	0
3043	1 (1.3)	1 (2.2)	0	1 (1.8)	0	2 (33.3)	0	0	1 (1.4)	0	1 (2.6)	0	1 (2.9)
Total	75 (100)	45 (60)	30 (40)	55 (73.3)	20 (26.7)	3 (4)	72 (96)	3 (4)	72 (96)	37 (49,3)	38 (50.7)	40 (53.3)	35 (46.7)

Results are expressed as a number (percentage). CZA; ceftazidime–avibactam, C/T; ceftolozane–tazobactam, R; resistant, S; susceptible.

**Table 2:** The frequency of association between different β-lactamase classes and resistance to ceftazidime–avibactam and ceftolozane–tazobactam among 75 MDR-*P. aeruginosa* isolates collected from Hamad Medical Corporation between 2014–2017.

Antibiotic	β-Lactamase Class	Resistant	Susceptible	*p*-Value *
		*N* = 37	*N* = 38	
Ceftazidime–avibactam	Class A	48.6%	31.6%	0.131
	Class B	54.1%	0%	<0.001
	Class C	100%	92.1%	0.240 ^†^
	Class D	91.9%	100%	0.115 ^†^
Ceftolozane–tazobactam		***N* = 40**	***N* = 35**	
	Class A	55%	22.9%	0.005
	Class B	50%	0%	<0.001
	Class C	95%	97.1%	0.99 ^†^
	Class D	92.5%	100%	0.243 ^†^

* *p*-value was calculated using Pearson Chi-Square test, ^†^ *p*-value was calculated using Fisher Exact test.

**Table 3:** The distribution of different β-lactamase genes associated with their minimum inhibitory concentration (MIC) values of ceftazidime–avibactam and ceftolozane–tazobactam.

**Antimicrobial Agent**	**Ceftazidime–Avibactam**			**Ceftolozane–Tazobactam**			**Total Genes**
Susceptibility Result	Susceptible	Resistant	Extremely Resistant	Susceptible	Resistant	Extremely Resistant	
	MIC 0.75–8	MIC 12–192	MIC ≥ 256	MIC 0.75–8	MIC 12–48	MIC ≥ 256	
Gene	Frequency (%)			Frequency (%)			
Class A β-lactamase							
VEB-9	3 (15.8)	10 (52.6)	6 (31.6)	0	1 (5.6)	18 (94.7)	19
TEM-116	6 (100)	0	0	5 (83.3)	1 (16.7)	0	6
CTX-M-15	2 (66.7)	0	1 (33.3)	2 (66.7)	0	1 (33.3)	3
SHV-11	1 (33.33)	1 (33.33)	1 (33.33)	1 (33.33)	1 (33.33)	1 (33.33)	3
TEM-1	0	0	1 (100)	0	0	1 (100)	1
TEM-126	0	0	1 (100)	0	0	1 (100)	1
CARB-3a	1 (100)	0	0	1 (100)	0	0	1
*p*-value ^†^		0.002			<0.001		
Class B β-lactamase							
VIM-2	0	14 (87.5)	2 (12.5)	0	3 (18.7)	13 (81.3)	16
IMP-1	0	0	3 (100)	0	0	3 (100)	3
VIM-5	0	0	2 (100)	0	0	2 (100)	2
*p*-value ^†^		0.001			0.99		
Class C β-lactamase							
PDC-3	11 (47.8)	5 (21.7)	7 (30.4)	10 (43.5)	1 (4.3)	12 (52.2)	23
PDC-35	0	13 (86.7)	2 (13.3)	0	0	15 (100)	15
PDC-7	4 (40)	6 (60)	0	3 (30)	2 (20)	5 (50)	10
PDC-1	7 (77.8)	1 (11.1)	1 (11.1)	8 (88.9)	0	1 (11.1)	9
PDC-5	5 (100)	0	0	4 (80)	1 (20)	0	5
PDC-10	5 (100)	0	0	5 (100)	0	0	5
PDC-8	1 (33.33)	1 (33.33)	1 (33.33)	2 (66.7)	0	1 (33.33)	3
PDC-9	1 (100)	0	0	1 (100)	0	0	1
*p*-value ^†^		<0.001			<0.001		
Class D β-lactamase							
OXA-50	20 (64.5)	4 (12.9)	8 (25.8)	19 (59.4)	2 (6.2)	11 (34.4)	32
OXA-488	12 (42.8)	15 (53.6)	2 (7.14)	10 (34.5)	0	19 (56.5)	29
OXA-10	3 (16.7)	7 (38.9)	8 (44.4)	1 (5.6)	1 (5.6)	16 (88.9)	18
OXA-486	6 (54.5)	4 (36.4)	1 (9.1)	6 (54.5)	1 (9.1)	4 (36.4)	11
OXA-4	0	4 (57.1)	3 (42.9)	0	1 (14.3)	6 (85.7)	7
OXA-114a	1 (25)	2 (50)	1 (25)	0	0	4 (100)	4
OXA-17	0	3 (100)	0	0	0	3 (100)	3
*E. coli* ampH	1 (50)	0	1 (50)	1 (50)	0	1 (50)	2
OXA-14	0	1 (100)	0	1 (100)	0	0	1
OXA-129	0	0	1 (100)	0	0	1 (100)	1
LCR-1	0	0	1 (100)	0	0	1 (100)	1
*p*-value ^†^		<0.001			0.001		
Total of genes	90 (38.3)	91 (38.7)	54 (23)	80 (34)	15 (6.4)	140 (59.6)	235
Total No. of isolates	38 (50.6)	26 (34.7)	11 (14.7)	35 (46.7)	5 (6.6)	35 (46.7)	75

Results are expressed as number (percentage), ^†^ *p*-value was calculated using the Fisher Exact test to examine the association between the β-lactamase gene and susceptibility patterns (susceptible, resistant, extremely resistant) to ceftazidime–avibactam and ceftolozane–tazobactam.

## Data Availability

The data presented in this study are available as Supplementary Material published together with this article or available from the authors upon request.

## Supplementary Materials

The following are available online at https://www.mdpi.com/article/10.3390/antibiotics11020130/s1. Table S1: Antimicrobial susceptibility of 75 MDR-*P. aeruginosa* isolates collected from Qatar between October 2014 – September 2017. Table S2: Clinical diagnosis and demographic profile of 42 patients with MDR-*P. aeruginosa* infections at participating Qatar hospitals.
