# Supplementary material for: Association of blaVIM-2, blaPDC-35, blaOXA-10, blaOXA-488 and blaVEB-9 β-Lactamase Genes with Resistance to Ceftazidime–Avibactam and Ceftolozane–Tazobactam in Multidrug-Resistant Pseudomonas aeruginosa
Source: Antibiotics (Basel). 2022 Jan 19;11(2):130. doi: 10.3390/antibiotics11020130 (PMC8868128; doi:10.3390/antibiotics11020130)
Supplement: Supplementary file 1 [file antibiotics-11-00130-s001.zip › antibiotics-1489514-supplementary.pdf]

Supplementary material

Association of *bla*$_{VIM-2}$, *bla*$_{PDC-35}$, *bla*$_{OXA-10}$, *bla*$_{OXA-488}$ and *bla*$_{VEB-9}$ β-lactamase genes with resistance to ceftazidime/avibactam and ceftolozane/tazobactam in multidrug-resistant *Pseudomonas aeruginosa*

Mazen A. Sid Ahmed [1,2], Faisal Ahmad Khan [2], Hamad Abdel Hadi [3,4], Sini Skariah [5], Ali A. Sultan [5], Abdul Salam [6], Abdul Latif Al Khal [3,4], Bo Söderquist [7], Emad Bashir Ibrahim [1,5], Ali S. Omrani [3,4] and Jana Jass [2,*]

[1] Department of Laboratory Medicine and Pathology, Microbiology Division, Hamad Medical Corporation, Doha, Qatar.

[2] The Life Science Centre - Biology, School of Science and Technology, 701 82 Örebro University, Orebro, Sweden.

[3] Communicable Diseases Center, Hamad Medical Corporation, Doha, Qatar.

[4] Division of Infectious Diseases, Department of Medicine, Hamad Medical Corporation, Doha, Qatar

[5] Department of Microbiology and Immunology, Weill Cornell Medicine-Qatar, Doha, Qatar.

[6] Department of Epidemiology and Biostatistics, King Fahad Specialist Hospital, Dammam, Saudi Arabia

[7] School of Medical Sciences, Faculty of Medicine and Health, Orebro University, 701 82 Örebro, Sweden.

* Corresponding author

Supplementary Table S1. Antimicrobial susceptibility of 75 MDR-*P. aeruginosa* isolates collected from Qatar between October 2014 – September 2017.

Isolate no.	ST	CST	GEN	TOB	AMK	CIP	MER	ATM	FEP	CAZ	CZA	C/T	DTR
4	17	S	R	R	R	R	S	R	R	S	S	S	No
154	27	S	R	S	R	S	R	R	R	R	S	R	No
428	179	R	R	R	R	R	R	R	R	R	R	S	Yes
51	233	S	R	R	R	R	R	S	R	R	R	R	No
199	233	S	R	R	R	R	R	S	R	R	R	R	No
220	233	S	R	R	R	R	R	R	R	R	R	R	Yes
508	233	S	R	R	R	R	R	S	R	R	R	R	No
527	233	S	R	R	R	R	R	S	R	R	R	R	No
1	235	S	R	R	S	R	R	R	R	R	R	R	Yes
9	235	R	R	R	R	R	R	R	R	R	R	R	Yes
26	235	S	R	R	R	R	R	R	R	R	R	R	Yes
27	235	S	R	R	S	R	R	R	R	R	R	R	Yes
37	235	S	R	R	R	R	R	R	R	R	R	R	Yes
99	235	R	R	R	R	R	R	R	R	R	R	R	Yes
128	235	S	R	R	R	R	R	R	R	R	R	R	Yes
129	235	S	R	R	R	R	R	R	R	R	R	R	Yes
131	235	S	R	R	R	R	R	R	R	R	R	R	Yes
134	235	S	R	R	R	R	R	R	R	R	R	R	Yes
143	235	S	R	R	R	R	R	R	R	R	R	R	Yes
169	235	S	R	R	R	R	R	R	R	R	R	R	Yes
176	235	S	R	R	R	R	R	R	R	R	R	R	Yes
203	235	S	R	R	R	R	S	R	R	S	S	R	No
209	235	S	R	R	R	R	R	R	R	R	R	R	Yes
250	235	S	R	R	R	R	R	R	R	R	R	R	Yes
36	244	S	R	R	R	R	R	R	R	R	R	R	Yes
126	244	S	R	R	R	R	R	R	R	S	S	S	No
86	252	S	S	S	S	S	R	R	R	R	S	S	No

190	253	S	S	S	S	S	R	8	R	S	S	S	No
5	274	S	R	S	R	R	R	R	R	S	S	S	No
32	274	S	S	S	S	R	R	R	R	S	S	S	No
110	274	S	R	S	R	R	R	R	R	S	S	S	No
161	274	R	S	S	R	R	R	R	R	S	S	S	No
123	292	S	R	R	R	R	S	S	R	R	S	S	No
11	308	S	R	R	R	S	R	R	R	R	R	R	No
12	308	S	R	R	S	R	S	R	R	R	R	R	No
98	308	S	R	R	R	R	S	R	R	R	S	R	No
349	310	S	R	S	R	S	R	S	R	S	S	S	No
78	313	S	R	S	R	R	R	S	S	S	S	S	No
263	348	S	S	S	R	R	R	S	R	R	S	S	No
16	357	S	R	R	R	R	R	R	R	R	R	R	Yes
17	357	S	R	R	R	R	R	R	R	R	R	R	Yes
40	357	S	R	R	R	R	R	R	R	R	R	R	Yes
41	357	R	R	R	R	R	R	R	R	R	R	R	Yes
130	357	S	R	R	R	R	R	R	R	R	R	R	Yes
135	357	S	R	R	S	R	S	R	R	S	S	R	No
208	357	S	R	R	R	R	R	R	R	R	R	R	Yes
212	357	S	R	R	R	R	R	R	R	R	R	R	Yes
10	381	R	S	S	S	R	R	R	S	S	S	S	No
19	389	S	R	R	R	R	R	R	R	S	S	S	No
140	389	S	R	R	R	R	R	R	R	R	S	S	Yes
175	389	S	R	R	R	R	R	R	R	R	S	S	Yes
253	389	S	R	R	R	R	R	R	R	R	S	S	Yes
311	389	S	R	R	R	R	R	R	R	R	S	S	Yes
350	389	S	R	R	R	S	R	R	R	R	S	S	No
323	446	S	R	S	R	S	R	R	R	S	S	S	No
142	560	S	S	S	S	S	R	R	R	R	S	S	No
447	598	S	S	S	S	R	R	R	R	R	S	S	Yes

180	639	S	S	S	S	R	R	S	R	S	S	S	No
66	664	S	R	R	R	R	R	R	R	R	R	R	Yes
420	699	S	S	S	S	R	R	R	R	R	R	S	Yes
498	773	S	R	R	R	R	R	S	R	R	R	R	No
125	823	S	R	R	R	R	R	S	R	R	R	R	No
183	823	S	R	R	R	R	R	S	R	R	R	R	No
200	823	S	R	R	R	R	R	S	R	R	R	R	No
241	1076	S	R	S	S	R	R	R	R	S	S	S	No
6	1284	S	R	R	S	R	R	R	S	R	S	S	No
20	1284	S	R	R	S	R	R	R	R	S	S	S	No
166	1284	S	R	R	S	R	R	R	R	S	S	S	No
187	1284	S	R	R	R	R	R	R	R	S	S	S	No
196	1284	S	R	R	R	R	R	R	R	R	S	S	Yes
207	2819	S	R	S	S	R	R	R	R	R	S	S	Yes
232	2819	S	S	S	S	R	R	S	R	R	S	S	No
119	3022	S	S	S	S	R	R	R	R	R	S	R	Yes
457	3043	S	S	S	S	R	S	R	R	S	S	S	No
148	1284	S	R	R	S	R	R	S	R	S	S	S	No
Total resistant isolates		6	62	54	54	67	68	60	72	54	37	40	35

AMK: amikacin; ATM: aztreonam; CAZ: ceftazidime; CIP: ciprofloxacin; CST: colistin; C/T: ceftolozane/tazobactam; CZA: ceftazidime/avibactam; DTR: difficult-to-treat resistance; FEP: cefepime; GEN: gentamicin; MDR: multidrug resistant; MEM: meropenem; MIC_{50}: minimum concentration at which 50% and of the isolates were inhibited; MIC_{90}: minimum concentration at which 90% and of the isolates were inhibited; R: resistance; S: susceptible; ST: sequence type; TOB: tobramycin.

Minimum inhibitory concentration (MIC) in mg/ml, shading indicates *P. aeruginosa* non-susceptibility to the corresponding antimicrobial agent. Clinical Laboratory Standards Institute (CLSI) breakpoints for susceptibility: CIP ≤1, ATM ≤8, FEP ≤8, MEM ≤2, CAZ ≤8, GEN ≤4, TOB ≤4, AMK ≤16, C/T ≤ 4, CZA ≤8 and CST ≤2 mg/ml.

DTR was defined as nonsusceptibility to all tested β-lactams, monobactams and fluoroquinolones, excluding β-lactams/β-lactamase inhibitor combinations (CZA and C/T) [1].

Supplementary Table S2. Clinical diagnosis and demographic profile of 42 patients with MDR-*P. aeruginosa* infections at participating Qatar hospitals.

Characteristics	Frequency (%)	Characteristics	Frequency (%)
**Hospital**		**Clinical diagnosis**	
HGH	38 (90.5)	Infection	22 (52.4)
RR	2 (4.8)	Colonization	20 (47.6)
HH	1 (2.4)	**Acquisition**	
NCCCR	1 (2.4)	Hospital acquired	40 (95.2)
**Location**		Community acquired	2 (4.8)
Inpatient	22 (52.4)	**Gender**	
ICU	6 (14.3)	Male	35 (83.3)
Outpatient	14 (33.3)	Female	7 (16.7)
**Infection isolation site**		**Age group**	
Urine	14 (33.3)	Paediatric < 14 years	3 (7.1)
Skin & soft tissue	10 (23.8)	Adult 14-65 years	29 (69.1)
Respiratory	9 (21.4)	Geriatric >65 years	10 (23.8)
Blood	8 (19)	Range	1.5-81 years
Catheter tip	1 (2.4)	**Nationality**	
**Strain mucoidity**		MENA	17 (40.5)
Non-mucoid	37 (88.1)	Qatari	15 (35.7)
Mucoid	5 (11.9)	Indian Subcontinent	9 (21.4)
		Western Country	1 (2.4)
**Total**	42		42

HGH; Hamad General Hospital, RH; Rumailah Hospital, NCCCR; National Center for Cancer Care and Research, HH; Heart Hospital.

MENA; Middle East and North Africa

Supplementary Material References

1. Kadri, S.S.; Adjemian, J.; Lai, Y.L.; Spaulding, A.B.; Ricotta, E.; Prevots, D.R.; Palmore, T.N.; Rhee, C.; Klompas, M.; Dekker, J.P.; Powers, J.H. 3rd; Suffredini, A.F.; Hooper, D.C.; Fridkin, S.; Danner, R.L.; National Institutes of Health Antimicrobial Resistance Outcomes Research Initiative (NIH–ARORI). Difficult-to-Treat Resistance in Gram-negative Bacteremia at 173 US Hospitals: Retrospective Cohort Analysis of Prevalence, Predictors, and Outcome of Resistance to All First-line Agents. *Clin Infect Dis*. **2018**, 67, 1803-1814.
